# Target-fueled DNA walker for highly selective miRNA detection†
†Electronic supplementary information (ESI) available: DNA strand structure and sequences, assembly of DNA strands as noted in the text. See DOI: 10.1039/c5sc02784e



**DOI:** 10.1039/c5sc02784e

**Published:** 2015-09-10

**Authors:** Lida Wang, Ruijie Deng, Jinghong Li

**Affiliations:** a Department of Chemistry , Beijing Key Laboratory for Microanalytical Methods and Instrumentation , Tsinghua University , Beijing 100084 , China . Email: jhli@mail.tsinghua.edu.cn

## Abstract

Artificial DNA motifs as architectural scaffolds have been widely used to assemble a variety of nanoscale devices. Synthetic DNA nanostructures have accomplished mechanical switching in response to external stimuli, suggesting the promise of constructing a walking device that is being used in the field of biosensors. Here, we design a novel miRNA-responsive DNA walker biosensor based on strand displacement cascades and an enzymatic recycling cleavage strategy. By using miRNA as a driving force, the DNA walkers can be activated to move along the track and generate specific signals for let-7a with a high signal-to-noise ratio. This biosensor exhibits excellent analytical performance toward the sensing of let-7a with great specificity for resolving one nucleotide variation and a detection limit of 58 fM. Such an ultraselective sensor shows that DNA nanostructures have great potential in providing platforms for applications in the fields of biosensing, clinical diagnostics and environmental sample analysis.

## Abstract 2

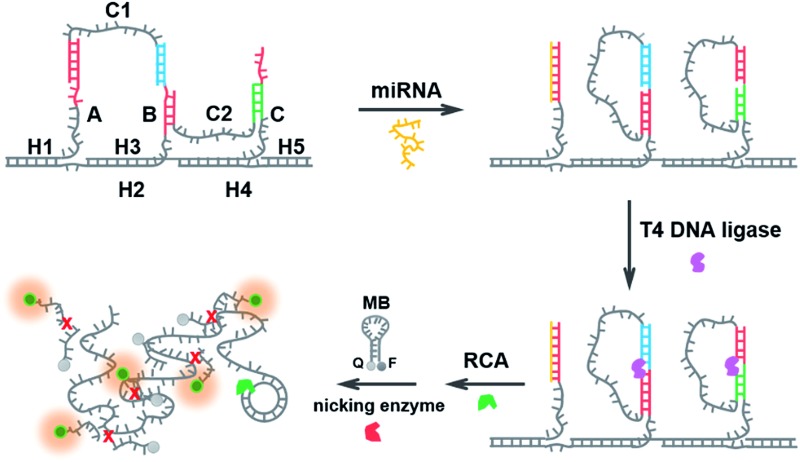
We report a DNA walking biosensor that can realize the detection of let-7a with a detection limit of 58 fM and high selectivity for resolving one nucleotide variation.

## Introduction

DNA is widely known as the carrier of genetic information and besides that, it has attracted substantial research interest for applications in the field of nanotechnology in recent years.^1–3^ DNA nanotechnology uses DNA strands as biological engineering materials to create static structures that perform mechanical operations such as DNA machines, including “DNA tweezers”, “DNA walkers”, *etc.* ^4–6^ Synthetic DNA machines have been developed to mimic important biological processes *in vitro*. In nature, a range of biological protein-based machines (myosin, kinesin and dynein) convert chemical energy from the hydrolysis of adenosine triphosphate (ATP) into mechanical work.^7–9^ Inspired by that, researchers paid more attention to using synthetic DNA strands as the building material to construct various DNA-based nanostructures. For example, H^+^/OH^–^, Hg^2+^ ions/cysteine or protein have been applied as external triggers for the running of the DNA machine.^10–12^ Gold nanoparticles and small molecules have been reported as cargos moving along the track in previous studies.^13–15^ In spite of the development of these methods, further improvement of the practical application is still in urgent need.

DNA walkers are synthetic DNA walking devices which consist of a biped and a well-defined track.^16,17^ Several DNA walking systems have been designed and constructed in a growing number of papers but rarely used in the field of biosensors.^18,19^ Incorporating DNA nanotechnology into biosensors remains in its early stages. Watson–Crick base pairs allow the purines to form hydrogen bonds to pyrimidines, with adenine (A) bonding to thymine (T) and uracil (U) in RNA, and cytosine (C) bonding only to guanine (G).^20^ Due to its specific recognition properties and structural features, DNA has been increasingly explored as a powerful and attractive building biomaterial for nanoconstruction. Compared to traditional materials, DNA shows great practical advantages, such as the predictability of their double helical structures, the simplicity of oligonucleotide synthesis and the excellent biological affinity.^21,22^ Therefore, using DNA as the architectural scaffolds, a DNA walker can have great potential for building up a sensitive and specific biosensor.

The movement of a DNA walker is based on a strand displacement cascade which is a process where an initiator strand displaces one pre-hybridized strand from a double helix.^23–25^ Strand displacement can be modulated with short complementary single-stranded segments of DNA (toeholds) through a branch migration process,^26–28^ which is complementary to a single-stranded DNA from a double helix. The displacement is driven by the selective binding affinity of the DNA substructures. Utilizing the toehold exchange-based strand displacement cascade as a key process, the DNA walker can be controlled by a specific target.

MicroRNAs (miRNAs) are small RNA sequences that mediate post-transcriptional regulation of specific gene expression.^29–31^ Recently, miRNA-based therapies have been widely exploited in cancer treatments.^32,33^ As many members within the same miRNA family share similar sequences and structures, it is still a challenge to distinguish between related miRNAs. let-7a is a typical member of a miRNA family that is associated with several cancers, such as lung and colon cancers. We want to demonstrate an ultrasensitive detection platform for microRNA by combining the recognition of miRNA and the tetrahedral DNA nanostructure. miRNA is used as the “fuel” to trigger the walker strand to move along the track and accomplish the conformational changes.

Rolling circle amplification (RCA) is an isothermal DNA amplification which produces long single-stranded DNA with repetitive units using a circularized template.^34,35^ A single circular molecule may be detected after RCA by a plethora of known analytical techniques which exploit the concatemeric repeats of the rolling circle product (RCP) as the detection sites.^36,37^ A molecular beacon (MB) as the signal output is complementary to the RCP. The DNA nicking enzyme, as a highly specific type of endonuclease, can recognize the particular double sequence between the MB and RCP, and cleave only the MB at the specific site to generate a fluorescence signal.^38,39^ RCA offers a more rapid, sensitive, specific and economical option.

In this study, a miRNA-responsive DNA walking biosensor based on miRNA-triggered strand displacement cascades was designed. The experiments demonstrated that the construction of a DNA walking bioassay was in response to miRNA. This walking bioassay was used for the sensitive detection of let-7a was due to the efficient rolling circle amplification reaction and the highly processive enzymatic recycling cleavage strategy. The assay can detect let-7a with a detection limit of 58 fM. The excellent specificity of clearly discriminating let-7a from closely related miRNAs due to the specific recognition properties of the DNA nanostructure. The construction of this DNA walker biosensor shows huge potential for further research to combine DNA nanotechnology with signal amplifying reporters to build sensing platforms.

## Experimental section

### Reagents and materials

T4 DNA ligase and phi29 polymerase (10 units per μL) were obtained from New England Biolabs (Beijing, China). Nb.Mva1269I (10 units per μL) was bought from Fermentas. Deoxyribonucleoside triphosphates (dNTPs, 100 mM), NaCl and KCl were bought from Beijing DingGuo Biotech. Co., Ltd. All solutions were prepared using ultrapure water. The DNA sequences were purchased from Shanghai Sangon Biological Engineering Technology & Services Co. Ltd. The molecular beacon (MB) modified with 5′-FAM and 3′-dabcyl was purified through high-performance liquid chromatography (HPLC). Other DNA sequences without modification were purified through denaturing polyacrylamide gel electrophoresis (PAGE). The sequences of the oligonucleotide probes used in this work are listed in Table S1 and Fig. S1.† Five single-stranded oligonucleotides (H1, H2, H3, H4, H5) were hybridized with each other to form the track with three protruding single-stranded branches (A, B, C). The sequences of the RCA products of C1 and C2 are complementary to the underlined sequences of the MB.

### Fabrication of the DNA walking biosensor

The walker was operated in the reaction buffer solution (33 mM Tris-acetate, pH 7.9 at 37 °C, 10 mM Mg-acetate, 66 mM K-acetate, 0.1% (v/v) Tween 20, 1 mM DTT) including H1, H2, H3, H4, H5, C1 and C2 in equal concentrations of 100 nM. The solution was incubated at 90 °C for 3 min, then slowly cooled at 65 °C for 30 min, 45 °C for 30 min, 37 °C for 30 min and 25 °C overnight to form the track with the folded C1 and C2.^18,40^

### miRNA driven movement and circularization

The movement of C1 and C2 along the track was activated by adding miRNA to the obtained solution. The mixture was incubated at 25 °C for 2 h. Then, a certain amount of T4 DNA ligase and 10× T4 ligase buffer (400 mM Tris–HCl, 100 mM MgCl_2_, 100 mM DTT, 5 mM ATP, pH 7.8 at 25 °C) were mixed into the reaction buffer. The reaction was incubated at 25 °C. The optimizations of the concentration of T4 DNA ligase and the reaction time were 0.1–10 units and 0–50 min, respectively.

### Rolling circle amplification (RCA) bioassay

The RCA reaction was carried out immediately after ligation. 1 μL of the RCA primer for C2, 5 μL of dNTPs (5 mM for each of dATP, dCTP, dGTP and dTTP; the final concentration of each dNTP was 500 μM), and 1 μL of phi29 DNA polymerase was added to each sample. The reaction mixture was incubated at 37 °C for a specified amount of time, and then heated at 90 °C for 10 min to stop the reaction and kept at room temperature.

### Fluorescence assay procedures

To conduct the fluorescence assay, 50 μL of 200 nM MB and a certain amount of Nb.Mva1269I were added to the prepared reaction mixture. After incubating at 37 °C for 45 min, the solution was transferred into 96 well plates and the fluorescence spectra were measured using EnVision Multilabel Plate Readers (Perkin-Elmer, USA). The excitation wavelength and emission wavelength were 495 nm and 520 nm, respectively.

### Instruments

The temperature was controlled using a PCR System (Bio-Rad, USA). Fluorescence spectra were monitored using the EnVision Multilabel Plate Readers (Perkin-Elmer, USA). Gel electrophoresis was performed on the prepared gel in TBE at 90 V for 30 min. After electrophoresis, the gel was visualized *via* a ChampGel 5000 (Beijing Sage Creation Science Co., Ltd, China).

## Results and discussion

### Configuration of the DNA walking bioassay

**Scheme 1:**
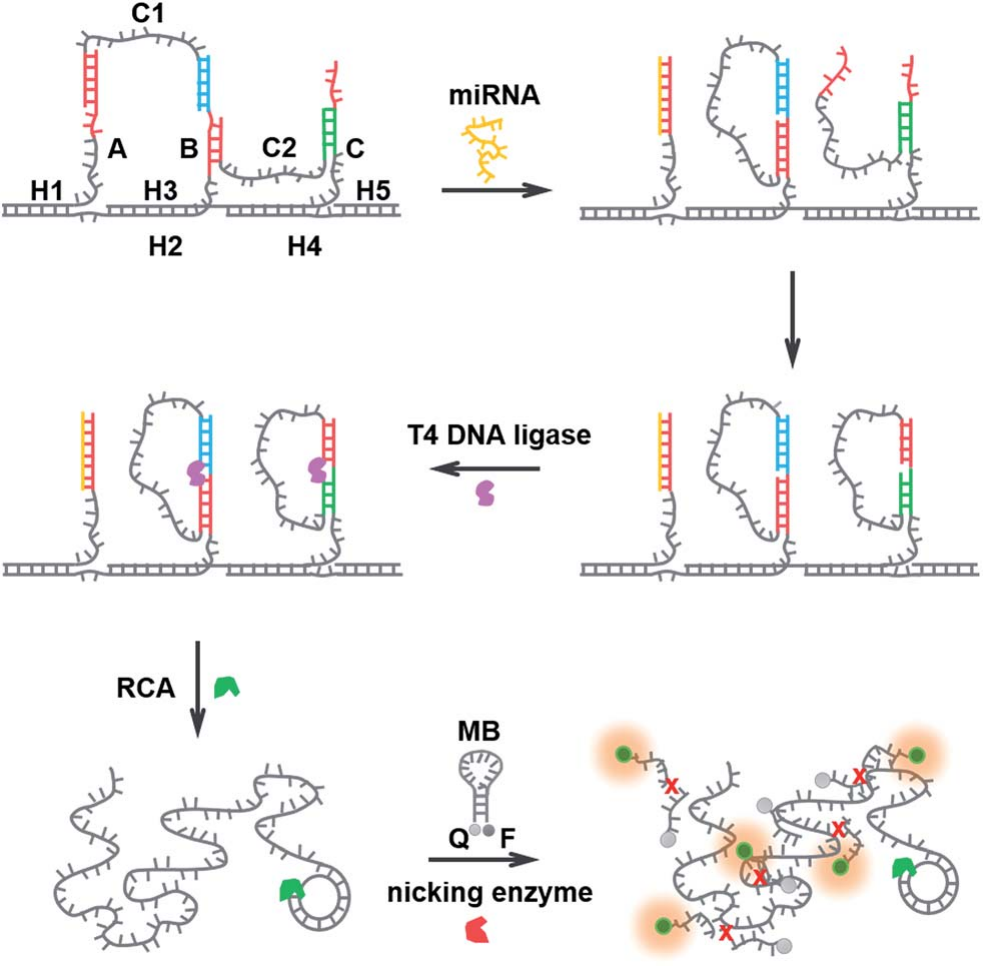
Schematic representation of the miRNA-responsive DNA walking nanostructure based on strand displacement cascades. (1) miRNA bound to branch A through toehold-mediated strand displacement, pushing the formed helices between the arm of strand C1 and the track to separate. (2) The released arm of strand C1 walked over to branch B, and the arm of strand C2 from branch B to branch C. (3) C1 and C2 were circulated using T4 DNA ligase. (4) The RCA reactions were performed. (5) The RCA product and the MB hybridized to each other, forming double-stranded DNA. The nicking enzyme catalyzed the multiple cleavage of the molecular beacon, resulting in the generation of a fluorescence signal.

The structural design of the DNA walker is shown in Scheme 1. Base sequences of all the oligonucleotides that make up the molecular device are shown in Fig. S2.† The miRNA-stimulated walking sensor is based on two components: a track and walker. The track, constructed of five oligonucleotides (H1, H2, H3, H4, H5), has three protruding single-stranded branches (A, B, C) separated evenly by scaffold helices. Single stranded C1 and C2 act as the walker. Both strands C1 and C2 consist of two arms, each reacts with one of the single-stranded branches of the track, and are spread out by the duplex. All positional transitions of the arms from one single-stranded branch to another along the track are performed using toehold-mediated strand displacement (TMSD). Conformational change in this biosensor built from oligonucleotides can be triggered by the addition of miRNA. miRNA specifically bound to the first branch, A, through TMSD, pushes the formed helices between the arm of strand C1 and the track to separate. This released arm of strand C1 walks over to branch B, serving as the input to the second strand displacement reaction, thus enabling the removal of the arm of strand C2 from branch B to branch C. Treatment of the system with miRNA results in the cascaded strand displacement reaction and generates two padlock probes. In the presence of T4 DNA ligase, these two padlock probes were circulated using branch B and C as the ligation templates. Then the DNA circles C1 and C2 acted as the circular templates to carry out the RCA reactions which generate long DNA strands containing tandem repeated sequences. The RCA products of C1 and C2 share the same sequence for the binding to the MB which is designed as the signal output unit. The MB also carries a specific recognition site for the nicking enzyme, Nb.Mva1269I.^41,42^ This nicking enzyme can recognize the specific sequence of the hybridized double-stranded DNA but only cleave the MB, resulting in the complete disconnection of the fluorophore from the quencher, and then a fluorescence signal appears. Thus the running of this miRNA-responsive DNA walker biosensor could be easily reflected by the fluorescence signal change.

### Detection capability for miRNA

The determination of let-7a in this method is realized through a strand displacement reaction, DNA ligation, RCA reaction and a nicking endonuclease-assisted fluorescence signal amplification. The fluorescence signal can reflect the running of this biosensor, so the concentration of miRNA can be calculated using the intensity of the fluorescence.

**Fig. 1:**
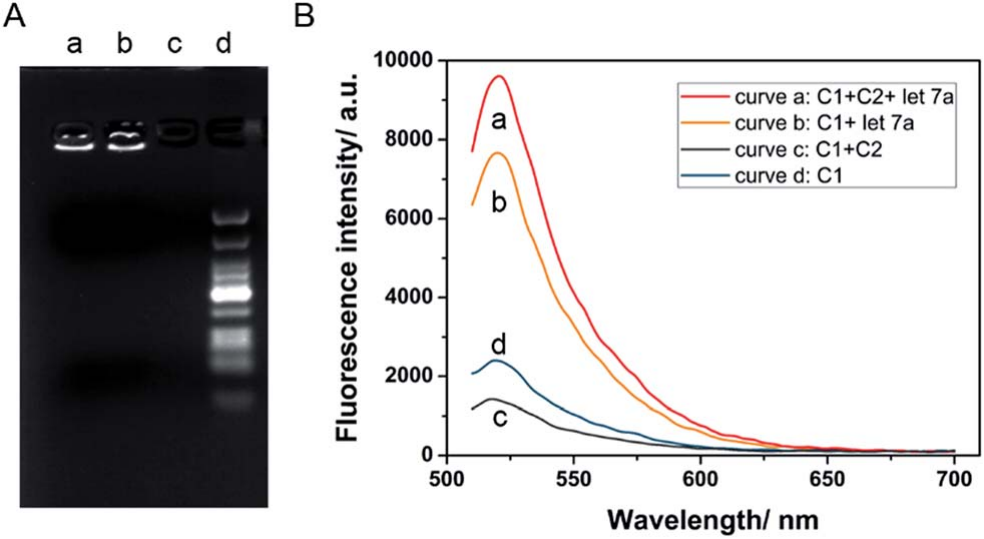
(A) Agarose gel with RCA products under different conditions: the DNA walking biosensor with only one walker, C1, incubated in the presence of let-7a (lane a); the DNA walking biosensor with two walkers, C1 and C2, incubated in the presence of let-7a (lane b) and in the absence of let-7a (lane c), and marker (lane d). (B) The fluorescence responses of the walking biosensor to 10 nM let-7a with background in the presence of the two walkers C1 and C2 (curve a and c), and in the presence of only walker C1 (curve b and d).

Initially, the ability of miRNA as an external trigger was assayed. The results of the reactions were analyzed using 1% agarose gel. As shown in Fig. 1A, when the DNA walking biosensors with one walker or two walkers were incubated with 10 nM let-7a, significant amounts of RCA products were observed (lane a and b). In contrast, less RCA product was produced when let-7a was replaced with dH_2_O (lane c). From the results, miRNA can be seen as the “fuel” and was able to activate the RCA reaction to generate long RCA products. let-7a miRNA showed great control over the biosensor. As expected, the DNA walking bioassay was able to utilize miRNA as the activator to catalyze the transfer of the walker from one branch to another along the track.

In order to assess the cascade reaction of the strand displacement, miRNA-induced fluorescence enhancements in the presence and absence of walker C1 and C2 were recorded. As shown in Fig. 1B, with only one walker, C1, the fluorescence signal in the presence of let-7a (curve b) was higher than the one without let-7a (curve d), but the fluorescence change was not large enough. On the contrary, by adding the second walker, C2, a more obvious signal enhancement in the fluorescence intensity was observed upon the addition of let-7a (curve a and c). By introducing the second walker, we observed an increased signal-to-noise ratio. The main reason for this fact is that the left arm of the second walker, C2, bound to branch B decreased the opportunity for the formation of C1 into a padlock structure, thus increasing the fluorescence intensity in the presence of let-7a and decreasing the background at the same time. Thus, the established DNA nanostructure is effective for the detection of let-7a.

### Optimization of the detection conditions

**Fig. 2:**
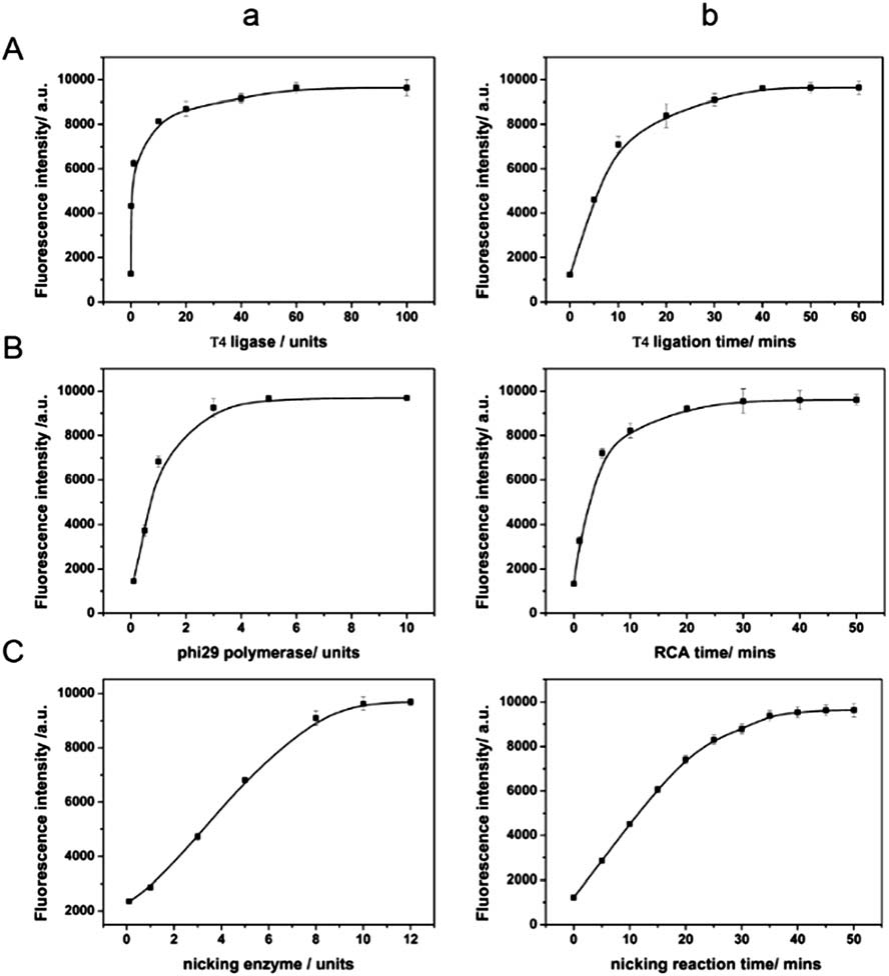
(A) Optimization of T4 DNA ligase concentration and time. (B) Optimization of phi29 DNA polymerase concentration and time. (C) Optimization of Nb.Mva1269I concentration and time. The assays were carried out in the reaction buffer, containing 10 nM let-7a, and 200 nM MB.

Various conditions were optimized to achieve the best miRNA responsive performance. First, the concentration and reaction time of T4 DNA ligase are the crucial parameters as the circularized DNA template generated by T4 ligation serves as the trigger for the next enzymatic recycling reaction. An insufficient quantity of T4 DNA ligase and an insufficient reaction time would make the fluorescence signal not remarkable enough. As can be seen from Fig. 2A(a), the fluorescence intensities increased along with T4 DNA ligase concentration, and reached the highest intensity at the concentration of 60 units indicating the complete ligation of the padlock form of DNA. In Fig. 2A(b), with the increase of incubation time, the fluorescence intensity increased at first, and then reached a maximum in 50 min. When the reaction time was longer than 50 min, the fluorescence signal barely changed. Therefore, 60 units and 50 min were chosen for the T4 DNA ligation process for further investigation. In addition, other significant factors such as the concentration of phi29 DNA polymerase and the incubation time were also studied. Because the fluorescence signal could be directly reflected by the RCA product, it is essential to optimize the conditions of the RCA reaction. As seen in Fig. 2B, the percent ligation values increased with the concentration and incubation time of phi29 DNA polymerase, and both reached a plateau at 5 units and 40 min, respectively. Both more enzyme and longer incubation time did not enhance it further. It indicates that the RCA reaction achieved saturation.

The sensing performance of the system is also closely related to the effect of the nicking enzyme, Nb.Mva1269I. Thus we further optimized the concentration and the incubation time in order to get the best performance, minimize the use of reagents and reduce the assay time. As can be seen from Fig. 2C(a), the fluorescence intensity of the sensing system was found to increase with the amount of Nb.Mva1269I and tend to a constant value at an amount of 10 units of Nb.Mva1269I. In Fig. 2C(b), the fluorescence intensity exhibited a rapid increase and then reached an equilibrium at 45 min. Thus, the optimized Nb.Mva1269I concentration and cleavage time were chosen to be 10 units and 45 min.

### Sensitivity and specificity of the detection

**Fig. 3:**
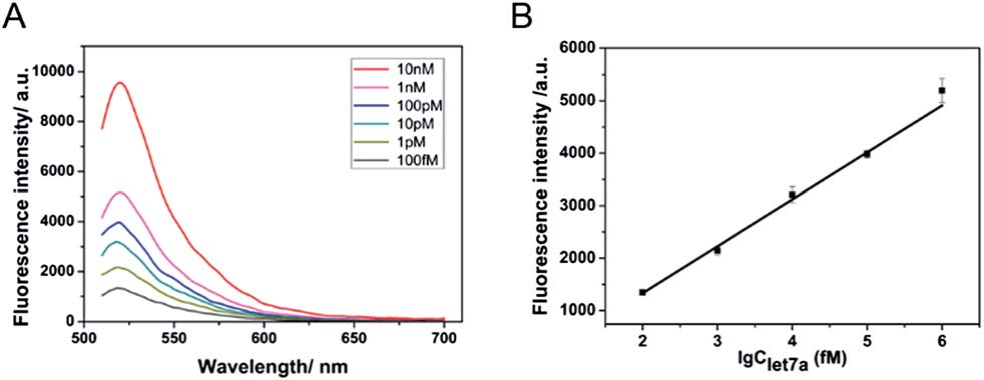
(A) Fluorescence detection of various concentrations of let-7a (top to bottom: 10 nM, 1 nM, 100 pM, 10 pM, 1 pM, 100 fM). (B) Dependence of the fluorescence intensity at 520 nm on the logarithm of let-7a concentration. The concentrations of Nb.Mva1269I and the MB were 10 units and 200 nM, respectively.

The proposed amplified sensing system is sensitive and specific to miRNA let-7a. To demonstrate the ability of the described DNA walking biosensor to sensitively quantify let-7a, a series of concentrations of let-7a (ranging from 100 fM to 10 nM) were measured based on the optimal assay conditions. Fig. 3A shows the fluorescence emission spectra of the sensing system upon the addition of let-7a at different concentrations. A gradual increase in fluorescence intensity was observed as let-7a concentration increased, thus indicating that the concentration of let-7a could be easily reflected by the change in the fluorescence signals. The fluorescence intensities and the logarithm of let-7a concentration exhibited a good linear relationship from 100 fM to 1 nM as shown in Fig. 3B. The detection limit (at 3 times the standard deviation of the background) was estimated to be 58 fM. This miRNA-responsive biosensor is very sensitive to let-7a.

**Fig. 4:**
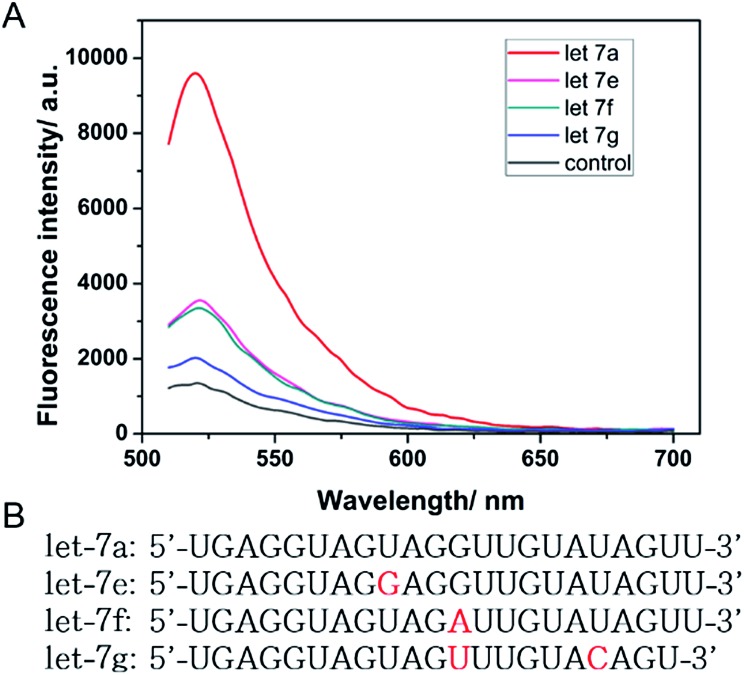
(A) Fluorescence intensity of the DNA walking bioassay driven by 10 nM let-7a, let-7e, let-7f and let-7g, respectively. The concentrations of Nb.Mva1269I and the MB were 10 units and 200 nM, respectively. (B) The sequences of let-7a, let-7e, let-7f, and let-7g. The bases that differ from those in let-7a are marked in red.

Besides the sensitivity, selectivity is another important issue to assess the performance of a sensor. Many miRNA family members have closely similar and identical sequences which pose extra difficulties when it is necessary to distinguish specific miRNAs.^28^ Therefore, an excellent selective biosensor is needed for distinguishing different miRNAs. To validate the selectivity of the described strategy, three similar and relevant let-7 family miRNAs, let-7e, let-7f and let-7g, were tested using the same reaction protocol described above. In detail, let-7e and let-7f contain one mismatch each and let-7g contains two mismatches. As shown in Fig. 4, the fluorescence intensity of let-7a was much higher than that of other miRNAs. The sensor exhibited great distinguishing ability as let-7a was induced as the specific “key” to the running of the walker. The strand displacement reaction was difficult to trigger with mismatches between the miRNAs and the DNA walker. The strand displacement reaction is a process based on the fact that the binding affinity of one strand and another pre-hybridized strand to a DNA template is different. Strand displacement can be modulated through the difference of the toehold.^26^ Therefore, the small differences between let-7a and the other three miRNAs resulted in very different fluorescence signals. Here, the results demonstrate that the biosensor is specifically triggered by let-7a. Fluorescence sensors reported in previous studies showed that the requirements of high selectivity for detection of miRNAs are growing rapidly and selective sensing remains a challenge. The high selectivity of our method reveals that it is comparable to many existing assay techniques for resolving cancer-related let-7 family members.^28,43^

## Conclusions

In summary, we have successfully utilized DNA nanotechnology to build a miRNA-responsive DNA walker biosensor. DNA has been regarded as a powerful and attractive building biomaterial for nanoconstruction owing to its specific recognition properties and structural features. In this work, a miRNA-responsive biosensor was developed on the basis of strand displacement cascades and an enzymatic recycling cleavage strategy. As only the target sequence (let-7a) can act as the “fuel” to meet the power need of the walker's movement along the track, this assay achieves stringent recognition for miRNAs, and is able to resolve miRNA family members with single-nucleotide variations. Additionally, rolling circle amplification and an enzymatic recycling cleavage strategy were applied to amplify the output of the DNA walker, achieving high sensitivity for miRNA detection. The positive results achieved here might become an initial guide for the potential applications of DNA nanotechnology. As DNA can be designed with various functions, the system in this work shows great potential in providing platforms for biosensing, clinical diagnostics and environmental sample analysis.
