# Supplementary material for: Target-fueled DNA walker for highly selective miRNA detection
Source: Chem Sci. 2015 Sep 10;6(12):6777–82. doi: 10.1039/c5sc02784e (PMC5508657; doi:10.1039/c5sc02784e)
Supplement: Supplementary file 1 [file SC-006-C5SC02784E-s001.pdf]

Electronic Supplementary Material (ESI) for Chemical Science.
This journal is © The Royal Society of Chemistry 2015

Electronic Supplementary Material (ESI)
This journal is © The Royal Society of Chemistry 2015

Electronic Supplementary Information

Target-fueled DNA walker for high selective miRNA detection

*Lida Wang, Ruijie Deng, and Jinghong Li**

Department of Chemistry, Beijing Key Laboratory for Microanalytical Methods and Instrumentation, Tsinghua University, Beijing 100084, China.

Electronic Supplementary Material (ESI)
This journal is © The Royal Society of Chemistry 2015

Table S1. Sequences of DNA probes and miRNAs in this work.

Name	Sequences (5'-3')
**H1**	5'- AAC TAT ACA ACC TAC TAC CTC ACT TTT ACG ACA CGG GTC AAG TCT AG -3'
**H2**	5'- CTA GAC TTG ACC CGT GTC GTG AGT GTG TGC AAT AGT TAG CTA TGA TGT CGT TAA CAA CCT ACT ACA GCA GCG AAT -3'
**H3**	5'- TAC TAC ACA TTC GTA CTT TGA CAG GAT CTA GTC TTG CCA TTA TGA CCA TCG ACA TCA TAG CTA ACT ATT GCA CAC ACT C -3';
**H4**	5'- ATG GTC ATA ATG GCA AGA CTA GAT CCT GTC CAT GCA ACT CGT AGC -3'
**H5**	5'- GCT ACG AGT TGC ATG -3'
**C1**	5'- p GTA GTA GGT TGT ATA GTT CAT CTT TAT TA<u>G AGG TGC ATT CAT</u> CAG TCC TTA GAT TCG CTG CT -3'
**C2**	5'- p GTA GTA GGT TAC TTT ATA TTA <u>GAG GTG CAT TCA T</u>CA GAT CTT AGG TAC GAA TGT -3'
**Primer-C2**	5'- CCT CTA ATA TAA AGT -3'
**MB**	5'6 -FAM-cga cga CTC <u>GAG GT ***GCATTC*** AT</u> AT tcg tcg -3' Dabcyl
**let-7a**	5'- UGA GGU AGU AGG UUG UAU AGU U -3'
**let-7e**	5'- UGA GGU AGG AGG UUG UAU AGU U -3'
**let-7f**	5'- UGA GGU AGU AGA UUG UAU AGU U -3'
**let-7g**	5'- UGA GGU AGU AGU UUG UAC AGU -3'

Lower-case letters represent the stem portion of MB. The underlined sequences of the MB are the same as the underlined sequences of C1 and C2, the RCA products of C1 and C2 are complementary to the underlined sequences of MB. The bold italic letters are the recognition sites of Nb.Mva1269I and lower-case letter 'p' indicates phosphate group.

Electronic Supplementary Material (ESI)
This journal is © The Royal Society of Chemistry 2015

DNA Strand Structure and Sequences.

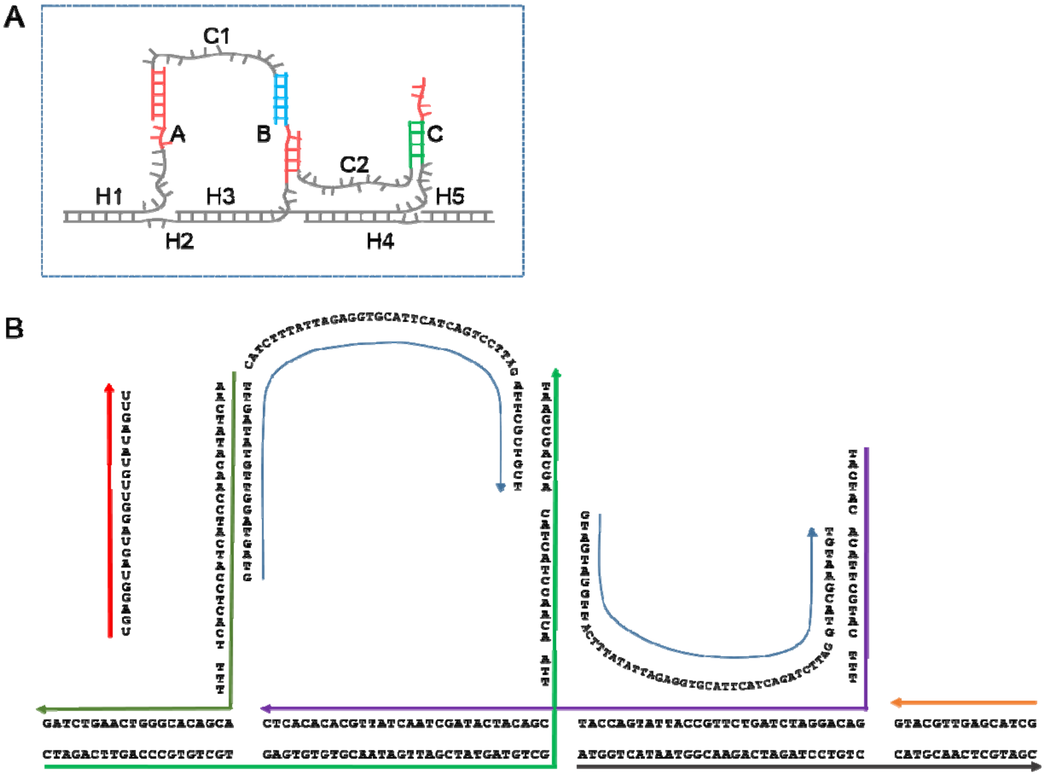

Fig. S1 (A) The structural design of the DNA walking biosensor. (B) Base sequences of the oligonucleotides that make up the DNA walking biosensor. The arrow indicate the direction of DNA.

The miRNA-stimuli walking device is based on two components: track and walker. The track, constructed of five oligonucleotides (H1, H2, H3, H4, H5), has three protruding single-stranded branches (A, B, C) separated evenly by scaffold helices. Each branches is positioned 30 base pairs away from its nearest neighbors and runs in opposite direction. Single-stranded branches provide flexibility for adopting different conformations. This distance allowed for the spatial control of the miRNA interaction. Single strand C1 and C2 are perform as the walker. Both Strand C1 and C2 consist of two arms, respectively and all of them react with the single-stranded branches of the track, and are spread out by duplex. C1, C2 and the track assemble together when each legs are attached to the three protruding single-stranded branches. All positional transitions of the arms from one single-stranded branches to another along the track are performed using toehold-binding, for which both the design of the track and the walker are endowed with appropriate DNA sequences centred around the structures of miRNA.

This journal is © The Royal Society of Chemistry 2015

Assembly of DNA Strands.

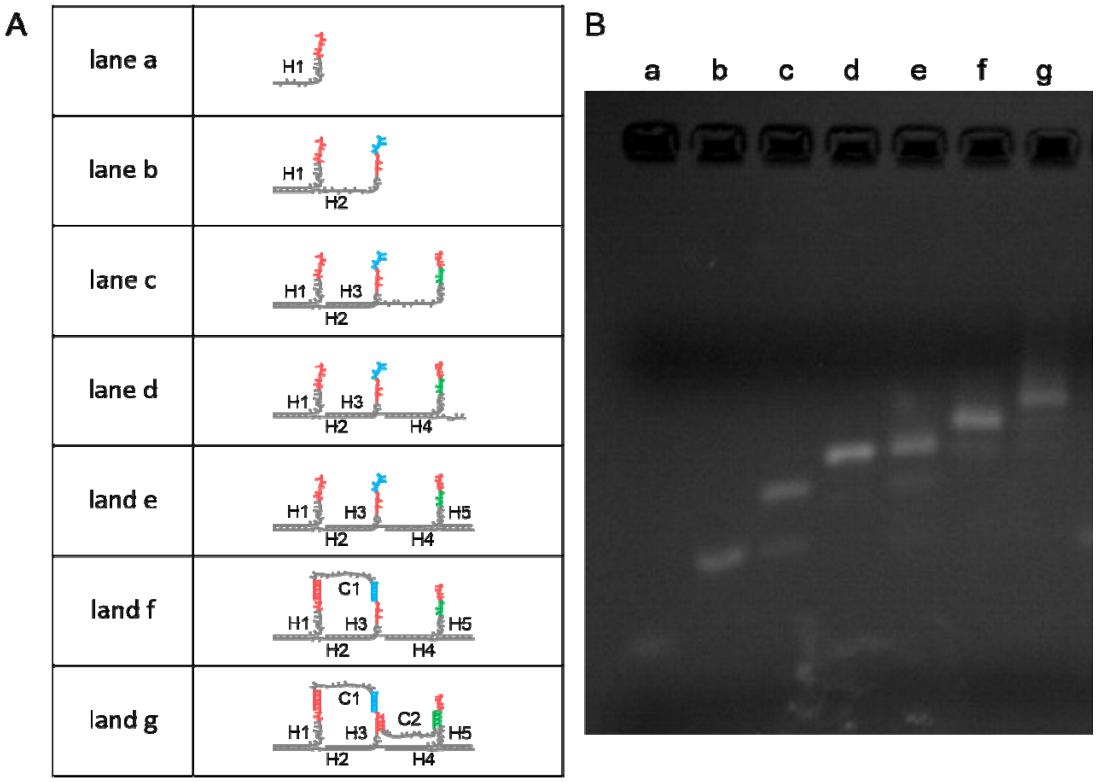

Fig. S2 (A) The form shows the different lane of the agarose gel represents different assembled DNA strands. (B) Agarose gel analysis of the result of self-assembly of DNA strands. The assemble of DNA strands was operated in reaction buffer solution (33 mM Tris acetate, pH 7.9 at 37℃, 10 mM magnesium acetate, 66 mM potassium acetate, 0.1% (v/v) Tween 20, 1 mM DTT) including different combination of H1, H2, H3, H4, H5, C1 and C2 at an equal concentration of 100 nM. The solution was incubated at 90℃ for 3 min, then slowly cooled at 65℃ for 30 min, 45℃ for 30 min, 37℃ for 30 min and 25℃ overnight to form the track with the folded C1 and C2.
